# Terminal hairpin in oligonucleotide dominantly prioritizes intramolecular cyclization by T4 ligase over intermolecular polymerization: an exclusive methodology for producing ssDNA rings

**DOI:** 10.1093/nar/gky769

**Published:** 2018-08-29

**Authors:** Yixiao Cui, Xutiange Han, Ran An, Yaping Zhang, Kai Cheng, Xingguo Liang, Makoto Komiyama

**Affiliations:** 1 College of Food Science and Engineering, Ocean University of China, Qingdao 266003, China; 2 Laboratory for Marine Drugs and Bioproducts, Qingdao National Laboratory for Marine Science and Technology, Qingdao 266003, China

## Abstract

When oligonucleotide bearing a hairpin near either its 3′- or 5′-end was treated with T4 DNA ligase, the intramolecular cyclization dominantly proceeded and its monomeric cyclic ring was obtained in extremely high selectivity. The selectivity was hardly dependent on the concentration of the oligonucleotide, and thus it could be added in one portion to the mixture at the beginning of the reaction. Without the hairpin, however, the formation of polymeric byproducts was dominant under the same conditions. Hairpin-bearing oligonucleotides primarily take the folded form, and the enzymatically reactive species (its open form) is minimal. As the result, the intermolecular reactions are efficiently suppressed due to both thermodynamic and kinetic factors. The ‘terminal hairpin strategy’ was applicable to large-scale preparation of a variety of DNA rings. The combination of this methodology with ‘diluted buffer strategy’, developed previously, is still more effective for the purpose. When large amount of l-DNA bearing a terminal hairpin (e.g. 40 μM) was treated in a diluted ligase buffer (0.1× buffer) with T4 DNA ligase, the DNA ring was prepared in 100% selectivity. Even at [l-DNA]_0_ = 100 μM in 0.1× buffer, the DNA ring was also obtained in pure form, simply by removing tiny quantity of linear byproducts by Exonuclease I.

## INTRODUCTION

**Figure 1.**
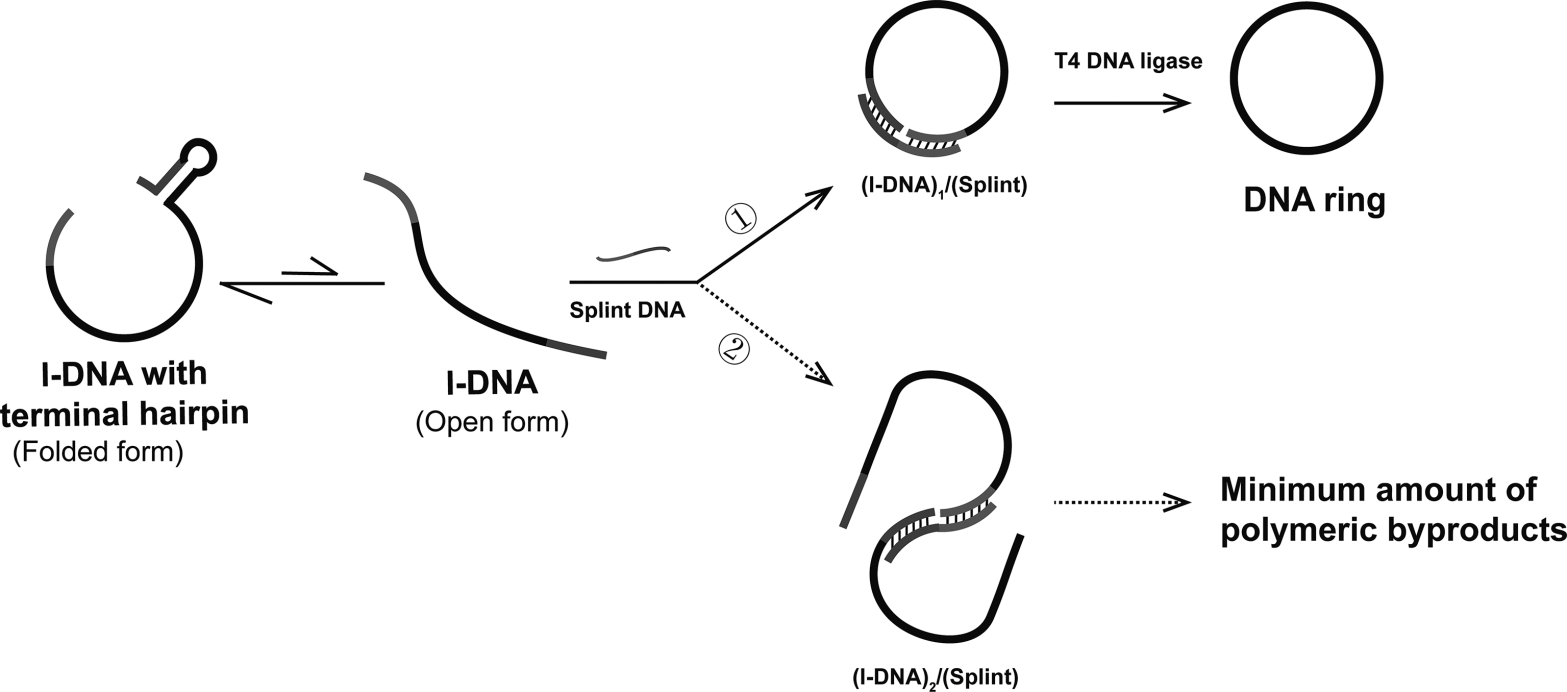
Outline of ‘terminal hairpin strategy’ for exclusive production of DNA rings. The single-stranded DNA (ssDNA) as active species for the ligation (the open form) is in equilibrium with l-DNA bearing a terminal hairpin (the folded form). This equilibrium is largely shifted towards the latter because of the steric requirements of hairpins so that the concentration of reactive ssDNA is sufficiently low to allow dominant occurrence of intramolecular ligation to DNA ring (pathway 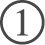 ).

Cyclic rings of single-stranded DNAs (c-DNA) are characterized by their unique features in mobility, dynamics, and topology, and one of the most important components in DNA nanotechnology (1–5). For example, DNA nanotubes were constructed by ssDNA rings and showed special ‘knitting nanoyarns’ phenomenon (6); molecular machines were designed by DNA rings with G-quadruplex and DNAzyme (7). Their valuable roles in molecular biology, medicine, and biotechnology have been also well documented (8–15). However, the methods to synthesize these attractive and promising nanostructures in a preparative scale have not been sufficiently established (16–19). In most cases, they are produced by intramolecular cyclization of a linear single-stranded DNA (l-DNA) with the use of ligases (e.g. T4 DNA ligase) (20–26). The ligation is promoted by adding splint, a short oligonucleotide which is complementary with the 3′- and the 5′-ends of l-DNA and places these ends in a close proximity in (l-DNA)_1_/(splint) complex (the upper middle in Figure 1).

The most crucial difficulty in large-scale synthesis of DNA rings is concurrent production of polymers of l-DNA as byproducts. The intermediates for both intramolecular cyclization and intermolecular polymerization are highly similar to each other (27,28). In most of previous syntheses of DNA rings, the concentration of l-DNA was kept very low throughout the reaction time (high dilution method), and intermolecular polymerization was minimized in terms of entropy difference. However, these reaction conditions are apparently incompatible with large-scale production of desired rings. A compromise solution to this problem (slow addition of l-DNA at controlled rates) is time-consuming, tedious, and furthermore needs sophisticated technique (29). Recently, an alternate solution to this problem (‘diluted buffer strategy’) was developed. Simply by decreasing the concentration of T4 DNA ligase buffer to abnormally low value (0.05–0.1× ligase buffer), the byproduct formation by T4 DNA ligase was greatly suppressed, and DNA rings were produced in high selectivity (30). In the diluted ligase buffers, the binding of l-DNA and splint by the enzyme is weakened, and the entropic difference between intramolecular and intermolecular reactions becomes more evident. In other words, the intermolecular ligation in diluted ligase buffers requires higher l-DNA concentration, compared with the reaction in the buffer of normal concentration. Dropwise addition of l-DNA to diluted buffers further improved the selectivity.

This paper presents much more convenient and versatile methodology for the production of DNA rings using T4 ligase, in which the entropic discrimination between intramolecular cyclization and intermolecular polymerization is further amplified by enthalpy factors (Figure 1). The key point is to place hairpin structure(s) in the vicinity of the joining ligation site (near either the 3′- or the 5′-end of l-DNA) and use it as selective suppressor of intermolecular polymerization. The l-DNA in solutions is in equilibrium between its folded form (less- or non-reactive; the left-most in Figure 1) and the open form (reactive for ligation; the second from the left in Figure 1). The open form is only the minor species so that the concentration of this reactive species is kept very low, even when l-DNA is directly added in one portion to the mixture and its concentration is large. Accordingly, ‘high dilution conditions’ are autonomously fulfilled, and intramolecular cyclization dominantly occurs to selectively provide the DNA ring (monomeric c-DNA). In terms of this ‘terminal hairpin strategy’, a variety of DNA rings are successfully prepared under conventional T4 DNA ligase conditions almost exclusively (around 100% selectivity, up to 20 μM). It is noteworthy that l-DNA applicable to the present strategy is easily designable for versatile DNA rings, since (i) the same ring is produced wherever the ligation site is located in the precursor l-DNA, and (ii) many single-stranded l-DNAs are intrinsically folded to secondary structures bearing hairpin(s) in solutions, as confirmed by our random screening experiments on human genome (*vide infra*). Compatibility of the present strategy with ‘diluted buffer strategy’ for eminent preparation of DNA rings is also evidenced.

## MATERIALS AND METHODS

### Materials

All oligonucleotides used in this study were purchased from GENEWIZ (Suzhou, China), and the sequences are listed in Supplementary Table S1. The secondary structures of linear DNAs (l-DNA) as the substrates were determined by Mfold calculation (31). Prior to the cyclization experiments, a phosphate was introduced to the 5′-position of l-DNA by using T4 polynucleotide kinase (Thermo Scientific; Pittsburgh, PA, USA). T4 DNA ligase, Exonuclease I and SYBR Green II were also obtained from Thermo Scientific (Pittsburgh, PA, USA). EvaGreen with the concentration of 20× was purchased from Biotium (Fremont, CA, USA). All other chemicals were from Sigma-Aldrich (St. Louis, MO, USA).

### Cyclization of single-stranded linear DNA (l-DNA)

A typical cyclization system (20 μl) contained l-DNA (5 μM), splint (10 μM) and T4 DNA ligase (10 U) in 1× T4 DNA ligase buffer ([ATP] = 500 μM, [MgCl_2_] = 10 mM, [DTT] = 10 mM, and [Tris–HCl] = 40 mM). In most of the cases, the ligation was performed at 25°C for 12 h and terminated by incubated the mixture at 65°C for 10 min. Single-stranded DNA rings were confirmed by Exonucleases I (10 U) at 37°C for 3 h. After the reactions, the products were subjected to 10% or 12% denaturing polyacrylamide gel (dPAGE) and stained with SYBR Green II.

### Evaluation of selectivity, yield and conversion

The data were analyzed by Image Lab software to quantify the fluorescence emission from each band. The calculating methods of selectivity and yield were the same as our last papers (30,34). The conversion for the formation of DNA rings were calculated by the following equation:

\begin{equation*}{\rm{Conversion}}\,\left( \% \right){\rm{ }} = {\rm{ }}(1 - \frac{R}{{P + C + R}}) \times 100,\end{equation*}

where *C, P* and *R* are the band intensities of circular single-stranded DNA ring, polymeric byproducts and remained single-stranded linear DNA (l-DNA), respectively. The results presented here are the averages of three independent experiments.

### Determination of *T*_m_ values

High resolution melting method (HRM) was used to determine the *T*_m_ of hairpins (32). The solutions of DNA (1 μM) were prepared in 1× T4 DNA ligase buffer containing EvaGreen (1×). The mixed oligomer solution (10 μl) was pipetted into 96-well microtiter plates and then transferred to a PikoReal Real-Time PCR instrument (Thermo Scientific, Finland). Annealing was performed with a cooling rate of 0.1°C/s from 95°C to 10°C; then, fluorescence data were collected over a temperature range of 10–95°C in 0.1°C increments (the holding time was 2 s). At least five parallel tests were carried out in one plate.

### Mfold calculation

The solution structures of l-DNAs in this study were simulated by The mfold Web Server using ‘DNA folding form’ (31). The typical conditions are: [Mg^2+^] = 10 mM, 25°C.

## RESULTS

**Figure 2.**
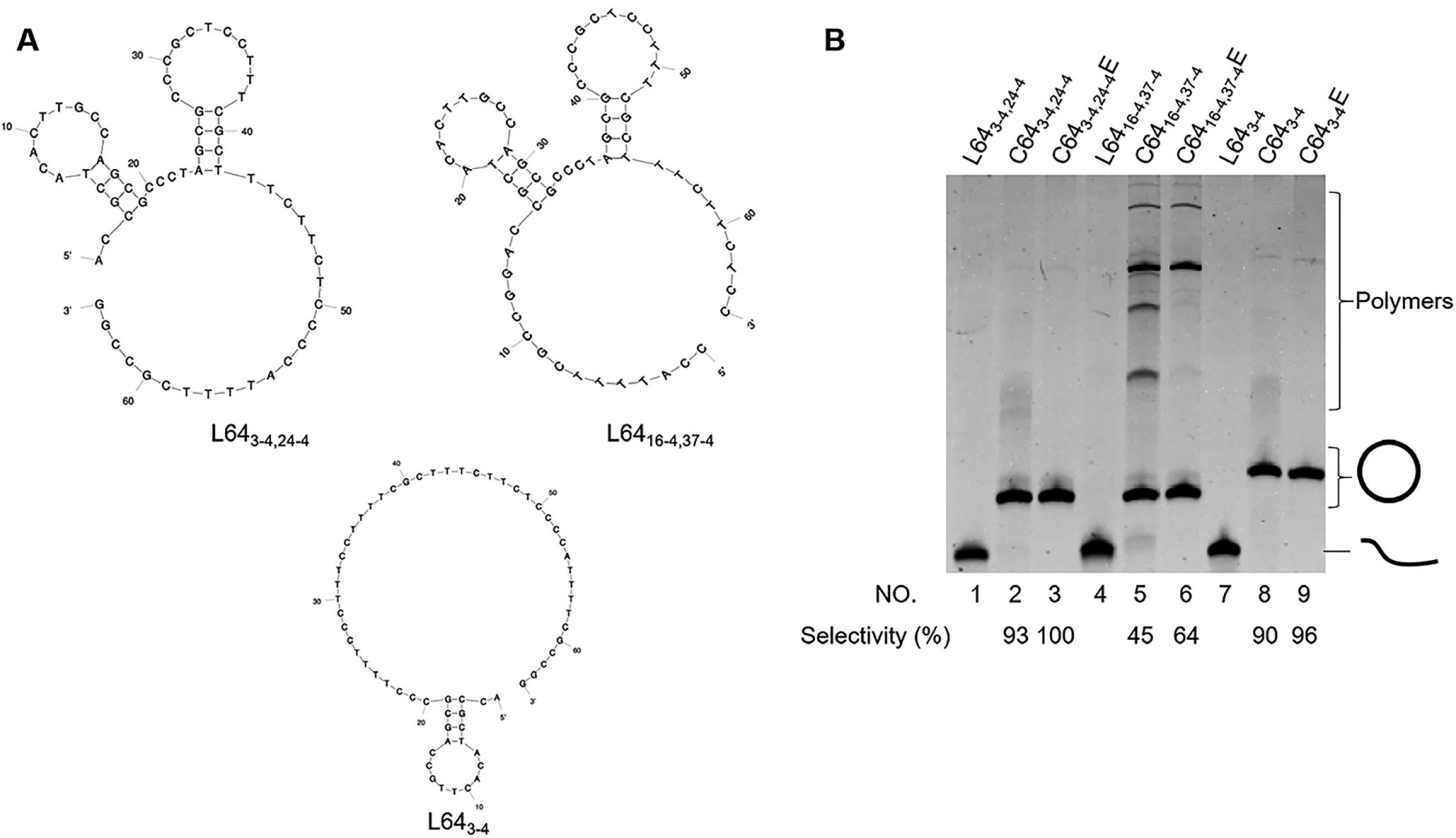
Dominant cyclization of l-DNA using hairpins as internal promoters. (**A**) The solution structures of L64_3-4,24-4_, L64_16-4,37-4_ and L64_3-4_, determined by Mfold calculation under the conditions of [Mg^2+^] = 10 mM and 25°C. (**B**) Treatments of these l-DNAs with T4 DNA ligase. Lane 1, L64_3-4,24-4_ without the T4 ligase treatment; lane 2, L64_3-4,24-4_ treated with T4 DNA ligase in the presence of 12-nt splint which is complementary with the 6-nt sequences in the 3′- and 5′-ends of L64_3-4,24–4_; lane 4, L64_16-4,37-4_ without the treatment; lane 5, L64_16-4,37-4_ treated with T4 DNA ligase in the presence of 12-nt splint. Lane 7, L64_3-4_ without the treatment; lane 8, L64_3-4_ treated with T4 DNA ligase in the presence of 12-nt splint. In lanes 3, 6 and 9, the products in lanes 2, 5 and 8 were further treated with Exonuclease I to remove non-cyclic products. The conditions for the T4 ligase reactions: [l-DNA]_0_ = 5 μM, [splint]_0_ = 10 μM and 10 U T4 DNA ligase in 1× T4 DNA ligase buffer at 25°C for 12 h.

In this paper, hairpins were placed at various places in linear oligonucleotides (l-DNAs), which were cyclized to DNA rings with the use of T4 DNA ligase at 25°C. The sizes and stabilities of hairpins in these l-DNAs, as well as their positions, were systematically changed. For example, L64_3-4,24-4_ in the upper left of Figure 2A is a 64-nt linear DNA, in which one hairpin involving 4-bp stem (and 11-nt loop) starts at the 3^rd^ nt from the 5′-end and another hairpin involving 4-bp stem (13-nt loop) starts at the 24^th^ nt. In this folded form, direct intramolecular cyclization should hardly occur, since its hybrid with a 12-nt splint is a 30-nt ring and difficult to be formed due to steric strain.

When the structures of l-DNAs were systematically changed, intense care was paid to maintain the whole conformation of oligonucleotides as much as possible. The cyclic structures of the target products, single-stranded DNA rings, were confirmed by their complete resistance against Exonuclease I.

### Remarkable promotion by a hairpin, adjacent to the ligation site, on T4 ligase-mediated intramolecular cyclization

When L64_3-4,24-4_ (Figure 2A, left top) was treated with T4 DNA ligase in the presence of a 12-nt splint, the intramolecular cyclization dominantly proceeded, and the corresponding c-DNA_64_ was almost exclusively obtained (lane 2 of Figure 2B). The conversion of L64_3-4,24-4_ to the ring was nearly quantitative. Here, at the beginning of the reaction, all the l-DNA used (5 μM) was added in one portion to the solution of T4 ligase and the splint. Even without using high dilution method, in which the concentration of l-DNA was always kept very low by its careful stepwise addition, the formation of polymeric byproducts was successfully suppressed by the hairpins. The cyclic structure of the product (the band designated by the circle) was confirmed by its complete resistance against Exonuclease I (lane 3). For the purpose of comparison, another 64-nt oligonucleotide L64_16-4,37-4_ (Figure 2A, upper right) was treated with T4 ligase reaction (lane 5), under exactly the same conditions as the reaction of L64_3-4,24-4_. The c-DNAs formed by these two l-DNAs have exactly the same sequence, and the difference is only the location of the ligation site. Accordingly, one of the two hairpins is located near the ligation site in L64_3-4,24-4_, whereas both hairpins are located far away from the ligation site in L64_16-4,37-4_. In the T4 reaction of L64_16-4,37-4_, however, the formation of the ring is far less efficient and significant amounts of polymeric byproducts (a series of bands in the upper part in the gel) were produced (compare lane 5 with lane 2 for L64_3-4,24-4_). The selectivity for the formation of c-DNA_64_ was only 45%. Note that the final product in these two reactions is exactly the same. Enormous effects of hairpins to promote the intramolecular cyclization are evident.

In L64_3-4_ (the bottom in Figure 2A), one of the hairpins in L64_3-4,24-4_ was removed, and only the hairpin near the 5′-end (the ligation site) was left. As shown by lane 9 in Figure 2B, the site-selectivity for the formation of c-DNA_64_ was 96%, which was close to the value of L64_3-4,24-4_. Thus, in the T4 reaction of L64_3-4,24-4_, the hairpin near the ligation site (the left one in Figure 2A) is directly responsible for the suppression of intermolecular polymerization and induces the selective formation of DNA rings. The mobility of C64_3-4_ is a little lower than that of C64_3-4,24-4_ and C64_3-4,37-4_. The probable reason is that the secondary structure can also form temporarily even in a denaturing condition. Independently, a hairpin (4-bp stem and 11-nt loop) was placed near the 3′-end of l-DNA (see Supplementary materials Figure S1). It was found that this hairpin is also very effective for selective cyclization of this l-DNA to the ring. All these results have confirmed that a hairpin near the ligation site of oligonucleotide (either in the 3′-side or in the 5′-side) greatly suppresses intermolecular polymerization by T4 DNA ligase and promotes its cyclization to monomeric ring. The validity of the proposal in Figure 1 has been substantiated.

**Figure 3.**
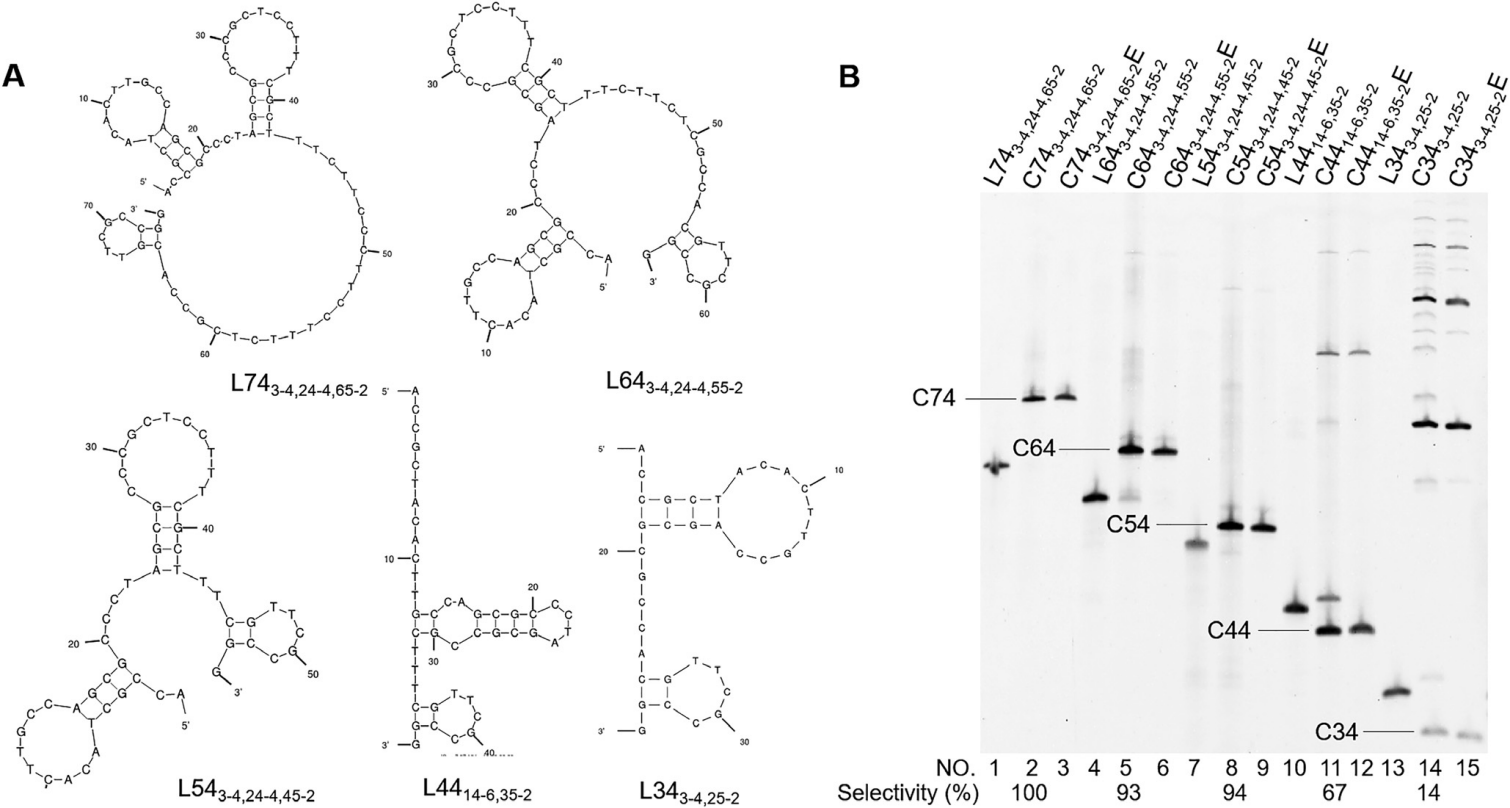
Terminal hairpin strategy for T4 DNA ligase-mediated preparation of DNA rings of smaller sizes. (**A**) Solution structures of 74-, 64-, 54-, 44- and 34-nt l-DNAs. (**B**) Gel electrophoresis patterns of the T4 ligase ligation products. The conditions of T4 ligase reactions are the same as described in Figure 2.

The ‘terminal hairpin strategy’ was also successfully applied to the preparation of DNA ring of various sizes (Figure 3). The DNA ring of 74-nt size was prepared almost in 100% selectivity. No polymeric byproducts were perceived (lane 2). Smaller rings of 64-nt and 54-nt sizes were also selectively obtained (selectivity = 93% in lane 5 and 94% in lane 8). The selectivity was not satisfactory, only when the length of l-DNA was still shorter (67% for 44-nt in lane 11 and 14% for 34-nt in lane 14). With this strategy, functional oligonucleotides which possess complicated tertiary structure (e.g. DNAzymes) can be also successfully cyclized in high selectivity (see Supplemental Figure S2).

### Factors governing the efficiency of terminal hairpin-promoted cyclization

#### Position of the terminal hairpin with respect to ligation site

**Figure 4.**
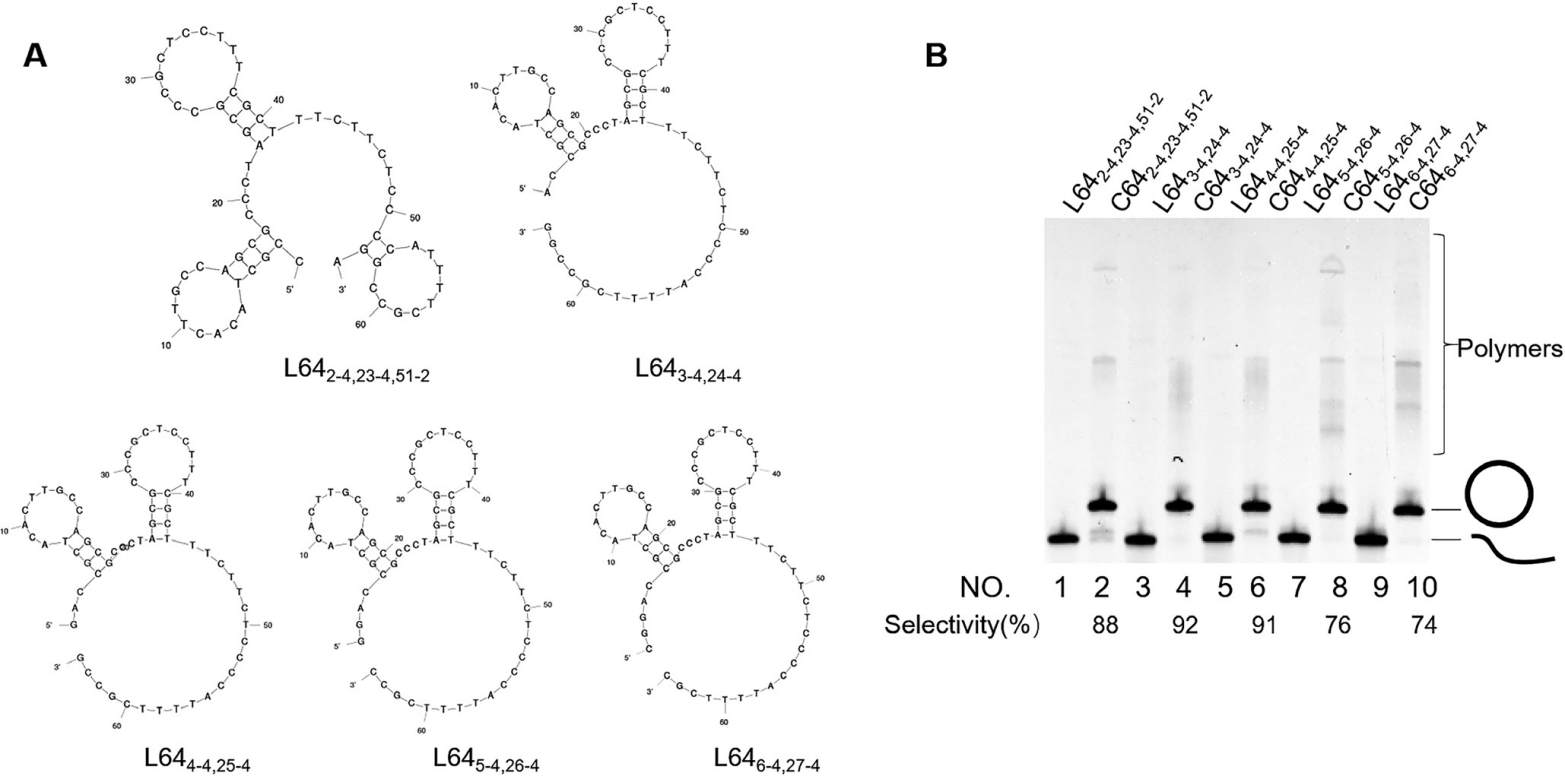
Effects of the distance between a hairpin and the ligation site on the magnitude of ‘terminal hairpin effect’ for the selective formation of single-stranded DNA ring. (**A**) The solution structures of l-DNAs used here. (**B**) Lane 1, L64_2-4,23-4,51-2_ without the treatment; lane 2, L64_2-4,23-4,51-2_ treated with T4 DNA ligase in the presence of 12-nt splint which is complementary with the 6-nt sequences in the 3′- and 5′-ends of l-DNA; lane 3, L64_3-4,24-4_ alone; lane 4, L64_3-4,24-4_ treated with T4 DNA ligase in the presence of 12-nt splint; lane 5, L64_4-4,25-4_ alone; lane 6, L64_4-4,25-4_ treated with T4 DNA ligase in the presence of 12-nt splint; lane 7, L64_5-4,26-4_ alone; lane 8, L64_5–4,26-4_ treated with T4 DNA ligase in the presence of 12-nt splint. lane 9, L64_6-4,27-4_ alone; lane 10, L64_6-4,27-4_ treated with T4 DNA ligase in the presence of 12-nt splint. Reaction conditions are the same as described in Figure 2.

In L64_3-4,24-4_ in Figure 2A, the terminal hairpin starts at the 3^rd^ nt from the ligation site (there exist two nucleotides between them). In order to evaluate how far the ‘terminal hairpin effect’ efficiently reaches, the length between the hairpin and the ligation site was systematically changed in Figure 4. When the numbers of nucleotides between them are 1, 2 and 3 (lanes 2, 4 and 6, respectively), the selectivities for the formation of the rings remained satisfactorily high (around 90%). The cyclization selectively proceeds by the terminal hairpin effect, as long as the distance to the ligation site is 3-nt or smaller.

With 4-nt and 5-nt distance, however, the selectivities considerably decreased to 76% (lane 8) and 74% (lane 10). Apparently, the hairpin was too far away from the ligation site. Direct analysis of the effect of other nucleotide numbers (–1 and 0) was not successful, since the corresponding change in the sequence of L64_3-4,24-4_ was accompanied by notable changes of the whole conformation of oligonucleotide, and a hairpin was formed at entirely different positions (see Supplemental materials, Figure S3)

#### Stability of the terminal hairpin

**Figure 5.**
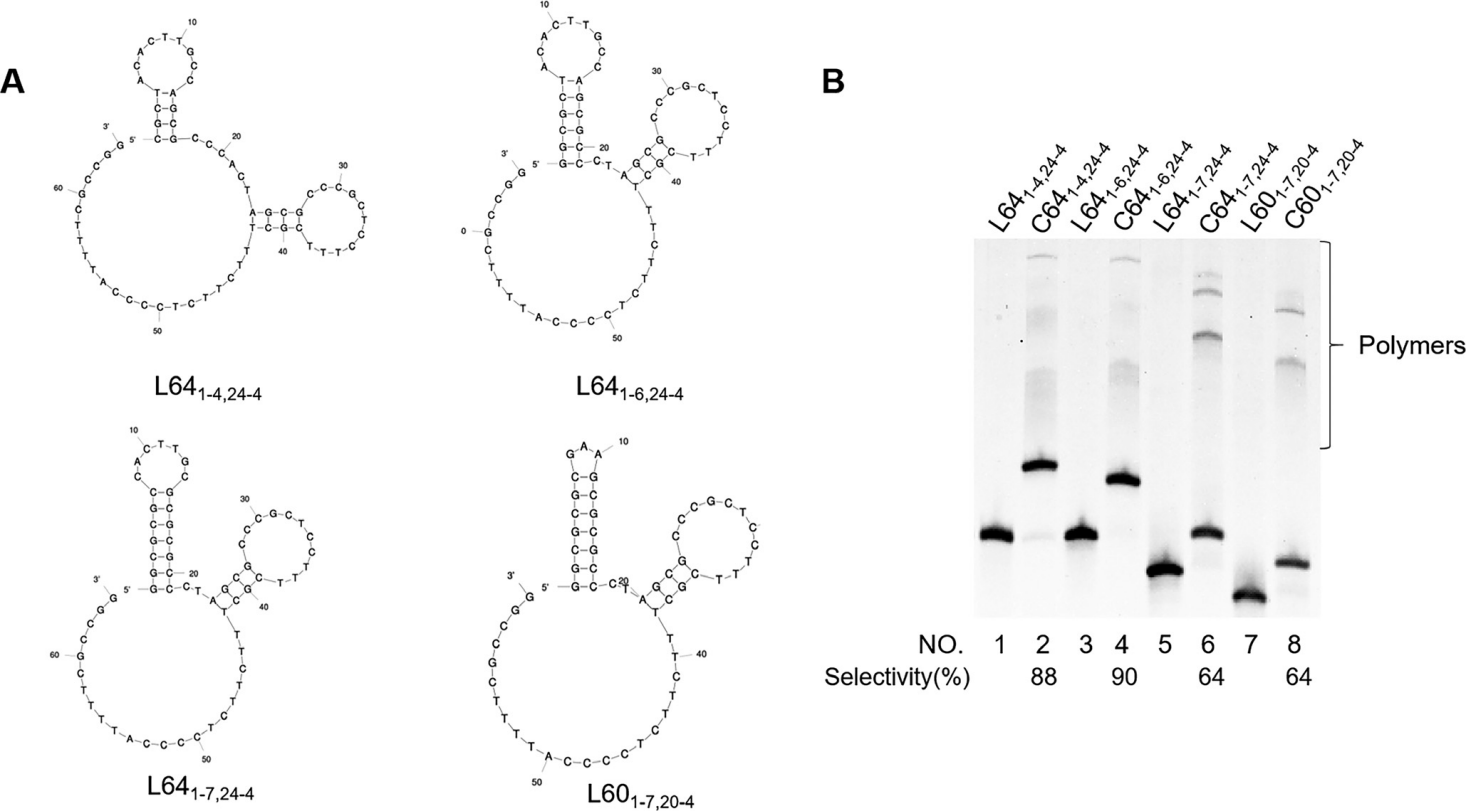
Effects of the stability of hairpin on the cyclization by T4 DNA ligase. (**A**) The solution conformations of L64_1-4,24-4_, L64_1-6,24-4_, L64_1-7,24-4_ and L60_1-7,20-4_, determined by Mfold calculation. (**B**) Lane 1, L64_1-4,24-4_ without T4 ligase treatment; lane 2, L64_1-4,24-4_ with T4 ligase treatment; lane 3, L64_1-6,24-4_ alone; lane 4, L64_1-6,24-4_ with T4 ligase treatment; lane 5, L64_1-7,24-4_ alone; lane 6, L64_1-7,24-4_ with T4 ligase treatment; lane 7, L60_1-7,20-4_ alone; lane 8, L60_1-7,20-4_ with T4 ligase treatment. The enzymatic conditions are the same as described in Figure 2.

In Figure 5, the stability of terminal hairpins near the 5′-end was systematically changed by altering the stem length (L64_1-4,24-4_, L64_1-6,24-4_, L64_1-7,24-4_, and L60_1-7,20-4_ in (A)). The *T*_m_s for the first three corresponding hairpins, determined in separate experiments, are 57, 73 and 85°C, respectively. For L60_1-7,20-4_ with a GAA loop, its *T*_m_ is even higher than 90°C (32,33). For these l-DNAs, the selectivities for the formation of c-DNA were 88, 90, 64 and 64%, respectively. In order to accomplish very high selectivity for the monomeric ring product, the hairpin should not be too stable, although its sufficient stability is certainly a requisite. Hairpin involving 4 or 6 bp stem are the most appropriate. Interestingly, even when the *T*_m_ of L64_1-7,24-4_ is 85°C, the cyclization can also occur, while the *T*_m_ of L64_1-7,24-4_/splint complex (5′-end, GGCGCG/CGCGCC, 3′-end, GGCCGC/GCGGCC) is lower than 40°C. The cyclization should be very efficient at an extremely low concentration of l-DNA of open form (Figure 1).

### Kinetic analysis on the terminal hairpin-promoted cyclization to pin down the origin of ‘terminal hairpin effect’

**Figure 6.**
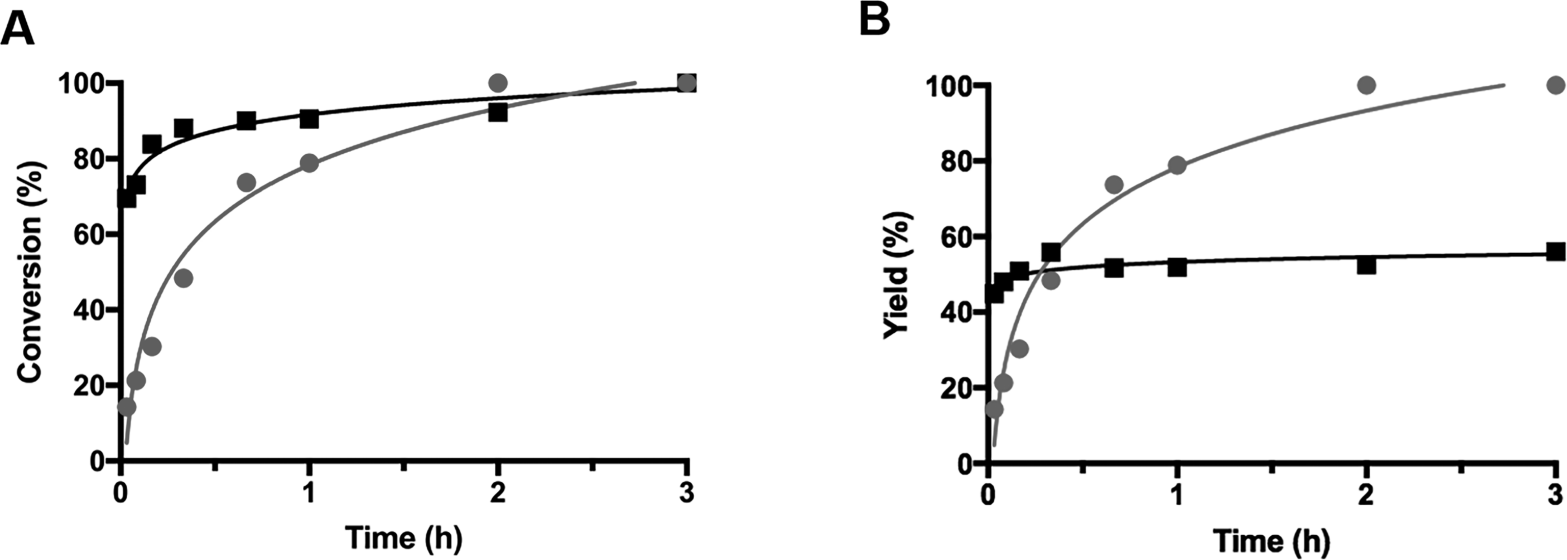
Comparison of the reaction conversion and yield of monomeric cyclic ring between substrates with and without the terminal hairpin. (**A**) Time-courses for the T4 ligase-mediated ligation of L64_3-4,24-4_ (circles) and L64_16-4,37-4_ (rectangles). The total amounts of DNA, consumed in the presence of T4 ligase (by both intramolecular and intermolecular ligation), are plotted as a function of reaction time. In (**B**), the yield of DNA ring is shown as a function of reaction time. Reaction conditions: [l-DNA]_0_ = 5 μM, [splint]_0_ = 10 μM, and 10 U T4 DNA ligase in 1× T4 DNA ligase buffer at 25°C.

As shown in Figure 6A, the ligation of L64_3-4,24-4_ bearing a hairpin near the ligation site is at least 10-fold slower than the ligation of L64_16-4,37-4_ (compare the circles with rectangles). However, it is noteworthy that almost all the L64_3-4,24-4_, consumed by the T4 ligation, was used for intramolecular ligation to form the cyclic product (the circles in Figure 6A and B almost completely superimpose each other). On the other hand, L64_16-4,37-4_ was more rapidly consumed, but more than half of the consumption was used for intermolecular ligation to the polymeric byproducts (compare the triangles in Figure 6A and B). The portion used for the DNA ring formation was only minor. Thus, ‘terminal hairpin effect’ for the selective cyclization is primarily ascribed to the suppression of the intermolecular reaction to polymeric byproducts (both linear and multi-cyclic). Exactly the same results were observed for other terminal hairpin-promoted selective cyclization to monomeric DNA rings (see Supplementary Figure S4).

### Minimal dependence of terminal hairpin-promoted cyclization selectivity on the concentration of linear DNA substrate, leading to selective and preparative-scale synthesis of DNA rings

**Figure 7.**
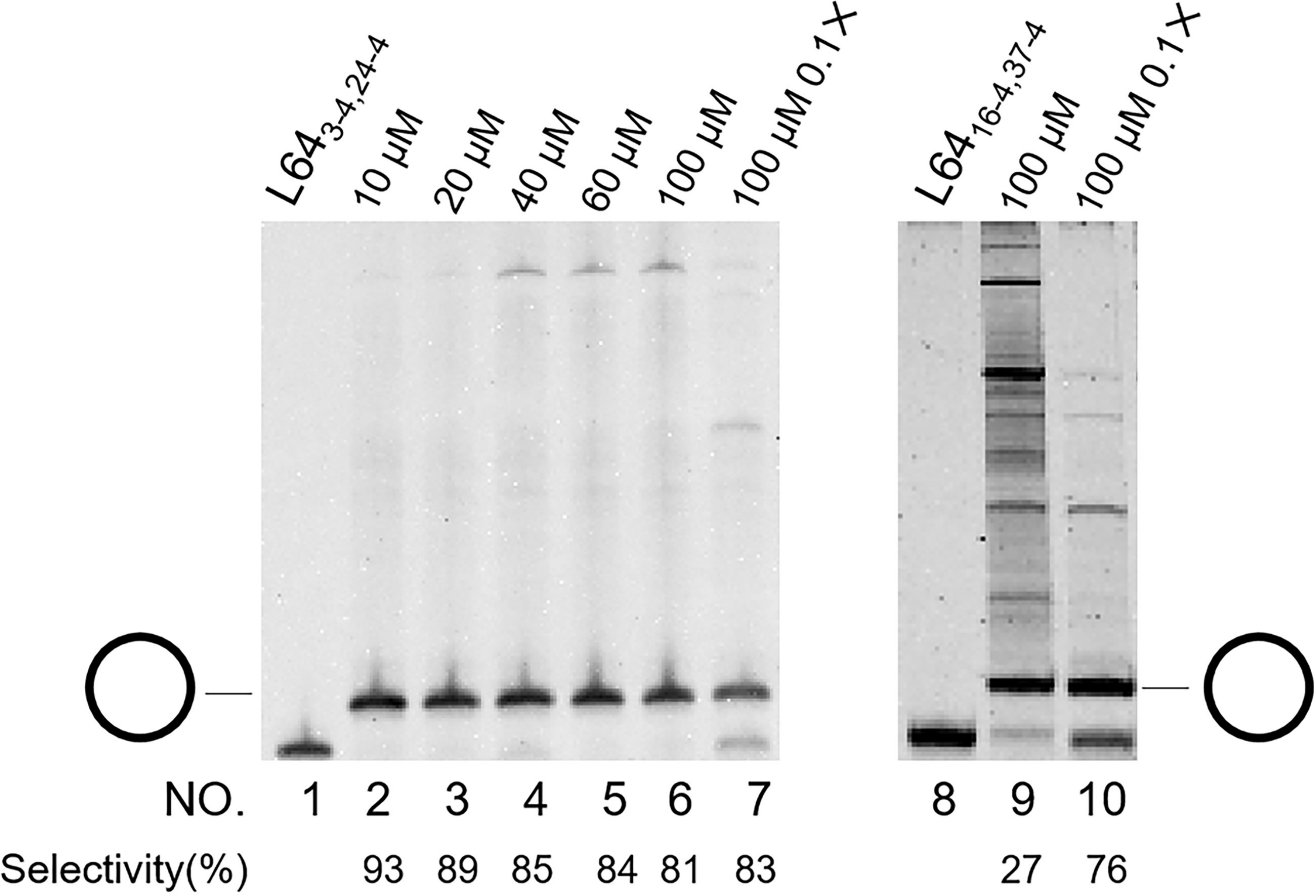
Highly selective cyclization at unusually high substrate concentrations using terminal hairpin strategy. Lane 1, L64_3-4,24-4_ without T4 treatment; lane 2, T4 reaction at [L64_3-4,24-4_]_0_ = 10 μM; lane 3, [L64_3-4,24-4_]_0_ = 20 μM; lane 4, [L64_3-4,24-4_]_0_ = 40 μM; lane 5, [L64_3-4,24-4_]_0_ = 60 μM; lane 6, [L64_3-4,24-4_]_0_ = 100 μM. In lanes 8_-_10, L64_16-4,37-4_ having no terminal hairpin is used. Lane 8, L64_16-4,37-4_ without T4 treatment; lane 9, [L64_16-4,37-4_]_0_ = 100 μM. Reaction conditions: [l-DNA]_0_/[splint]_0_ = 1/2 and 10 U T4 DNA ligase in 1 × T4 DNA ligase buffer at 25°C. In lanes 7 and 10, 0.1× T4 DNA ligase buffer was used in place of 1× T4 buffer, according to ref. (30) (see text for details).

In all the reactions presented above, the initial concentration of l-DNA substrates was 5 μM. It is noteworthy that the selectivity for the terminal hairpin-promoted cyclization is less dependent on the substrate concentration (Figure 7). When the concentration was 20 μM, the selectivity for the formation of c-DNA was 89% (lane 3), which was only slightly lower than the value at 10 μM. The selectivity was as high as 81%, even when [L64_3-4,24-4_]_0_ = 100 μM (lane 6). This result is highly in contrast with the enormous concentration effect for the reactions of L64_16-4,37-4_ which has no terminal hairpin. There, with increasing concentration of substrate, the formation of polymeric byproducts rapidly prevailed and became more dominant (compare lane 6 for L64_3-4,24-4_ with lane 9 for L64_16-4,37-4_). Apparently, the selectivity for this conventional DNA ligase reaction is strongly dependent on the substrate concentration, as was previously observed in many relevant reactions. The selectivity for the desired ring was only 27% at [L64_16-4,37-4_]_0_ = 100 μM.

In order to suppress intermolecular polymerization, with respect to targeted intramolecular cyclization, most of previous studies employed high dilution method where the concentration of l-DNA was kept very low. In order to keep the concentration of l-DNA minimal, l-DNA was dropwise added to the reaction mixture. This method requires much cost, time, and sophisticated technique, and still more critically preparation of sufficient amount of DNA ring is not easy. In the present ‘terminal hairpin method’, however, the selectivity is satisfactorily high even when large amounts of l-DNA are added in one portion to the reaction mixture and thus its concentration is notably high. Accordingly, sufficient amount of DNA ring can be easily prepared in preparative-scale simply by adding required amount l-DNA to the mixture at the beginning of the reaction. If we employ 1 mL reaction vessel, a few milligrams of DNA ring should be obtained. Further scale up is also easy due to simplicity of the reaction procedure. This is undoubtedly one of the most significant advantages of the present ‘terminal hairpin strategy’.

Significantly, the present strategy is completely compatible with ‘diluted buffer strategy’, developed in previous paper (30). By employing these two strategies simultaneously, highly pure specimens of monomeric DNA rings can be easily and straightforwardly prepared. When [L64_3-4,24-4_]_0_ = 100 μM and the T4 ligase reaction was achieved in 0.1× T4 ligase buffer, the selectivity was increased from the value (81%) in 1× buffer to 83% (compare lanes 6 and 7 in Figure 7). Still more crucially, in 1× T4 ligase buffer, considerable amounts of multiple cyclic products were produced as byproducts, together with linear polymers. Upon Exonuclease I treatment of the product from this conventional buffer to remove linear byproducts, the selectivity for the monomeric cycle was increased only to 86% (lane 5 in Supplementary Figure S5). In 0.1× buffer, however, almost no multiple cyclic products were formed during the T4 ligation. Thus, the monomeric cycle as the target product was obtained in 96% selectivity, after the T4 ligation product was simply treated with Exonuclease I (lane 10 in Figure S5). Superiority of the combination of ‘terminal hairpin strategy’ with ‘diluted buffer strategy’ was still more clearly evidenced, when [L64_3-4,24-4_]_0_ = 40 μM. By achieving the T4 ligase reaction of this mixture in 0.1× buffer, the monomeric DNA ring was directly synthesized in 100% selectivity without concurrent formation of any byproducts (lane 7 in Supplementary Figure S5). Post-treatment by Exonuclease I was never required here. This is highly in contrast with the formation of notable amounts of byproducts in 1× T4 ligase buffer (lane 3 in Supplementary Figure S5).

## DISCUSSION

The ‘terminal hairpin strategy’ is applicable to preparation of versatile DNA rings. As shown in (Supplementary Table S2), most of oligonucleotides involve one or more hairpins, when they are 50-nt or longer. Here, 100 single-stranded DNA fragments (50-nt or 100-nt) were randomly picked up from human genome, and analyzed by Mfold calculation. About 70% of all the 100-nt fragments involve one or more stable hairpins (*T*_m_ > 50°C, –Δ*G* > 3 kcal/mol), and the fraction of hairpin-bearing ones is also considerable (∼40%) even for 50-nt fragments. If slightly less stable hairpins are also acceptable for the present strategy, almost all these fragments have at least one hairpin. Accordingly, the preparation protocol of single-stranded DNA rings through ‘terminal hairpin strategy’ can be easily designed. First, the ring of desired sequence and size should be hypothetically cut at one site, and the resultant l-DNA should be analyzed by Mfold calculation to see whether a hairpin is located close to either the 3′- or the 5′-terminus of this l-DNA. Note that the hypothetical scission site is freely chosen, since all of the resultant l-DNAs provide the same DNA ring. The distance between the terminus of l-DNA and the start of the hairpin must be 1–3 nt, as evidenced by Figure 4. By repeating this process, the most appropriate l-DNA to prepare the targeted ring should be picked up. This l-DNA can be prepared by using a DNA synthesizer, and treated with T4 DNA ligase. Here, the l-DNA can be simply added to the reaction mixture in one portion. By this simple method, the target ring can be straightforwardly obtained in high selectivity. Although the reaction is retarded by the hairpin in this strategy, the reactions are sufficiently fast enough to attain satisfactorily high conversion within a few hours (see Figure 6).

The origin of the present terminal hairpin-induced selective synthesis of DNA rings is primarily the small concentration of the active species (the open form of the l-DNA bearing a terminal hairpin) in the ligation mixture. Because of the stability of the hairpin, the equilibrium between the open form and its inactive folded form is overwhelmingly shifted to the latter. For L64_3-4,24-4_ in Figure 2A, for example, Δ*G* of hairpin formation is –3.16 kcal/mol at 25°C, as estimated by Mfold calculation. The corresponding equilibrium constant (*K*) is 210. Accordingly, the concentration of the active species for the T4 ligase reaction (the open hairpin) is calculated to be only 0.024 μM, even when the concentration of l-DNA in the reaction mixture is 5 μM. For the reaction using 100 μM L64_3-4,24-4_, the concentration of the active species (the open hairpin) can be calculated to be 0.48 μM, which did not produce much polymeric byproducts (Figure 7). Thus, ‘high dilution conditions’, which have been widely employed for the intramolecular cyclization, are automatically fulfilled. The maximum preparative concentration for high selectivity (>85%) can be predicted by Δ*G* of hairpin formation (see Supplementary Figure S6). When the Δ*G* is –3.16 kcal/mol, for example, the predicted maximum concentration is about 20 μM (the concentration of the active species is set as 0.1 μM), which is consistent with our data (89% selectivity of 20 μM in Figure 7). Certainly, other factors should be considered such as DNA length, splint length, reaction temperature, etc.

In addition to this thermodynamic factor, a kinetic factor is also favorable to suppress the intermolecular reaction. Even when (l-DNA)_2_/(splint) complex is temporarily formed, one of these two l-DNAs is soon removed from the complex, since it is far more stable in its hairpin structure. As the result, mutual ligation of this complex to polymeric byproducts hardly occurs. Intermolecular binding of another l-DNA molecule by the unbound part of the splint is too inefficient under the conditions employed. On the other hand, (l-DNA)_1_/(splint) is sufficiently stable, and successfully lead to the cyclization. Even when either end of the l-DNA is removed from the splint, favorable entropy term allows its prompt rebinding.

Recently, we reported that secondarily structured oligonucleotides can be successfully cyclized by using thermostable *Taq* DNA ligase at high temperatures (34). The ‘terminal hairpin strategy’ in this study is a significant extension in that secondary structures of DNA are successfully used as gate keeper to hold on intermolecular polymerization which is a significant problem for effective preparation of DNA rings. Basically this new methodology can be used for cyclization of most sequences. The *Taq* DNA ligase may be used only for cyclization of DNA with several secondary structures, which is less efficient using this ‘terminal hairpin strategy’.

## CONCLUSION

For preparation of DNA rings by cyclization of oligonucleotides with T4 DNA ligase, a hairpin near the end of the oligonucleotide is enormously effective. Even when this hairpin-bearing oligonucleotide is added in one portion to the ligase buffer and otherwise intermolecular reactions are dominant, these side-reactions are satisfactorily suppressed by ‘terminal hairpin effect’. In these hairpin-bearing oligonucleotides, the concentration of the active species for the ligation (the open form) is minimized, providing favorable conditions for intramolecular cyclization over intermolecular polymerization. Most of oligonucleotides (>50 nt) involve hairpin(s), so that a variety of DNA rings can be selectively synthesized in terms of terminal hairpin strategy. Furthermore, the present strategy is satisfactorily compatible with ‘diluted buffer strategy’, which was previously developed in our laboratory (30), for preparative synthesis of monomeric DNA ring. When a required amount of l-DNA bearing a terminal hairpin (e.g. 100 μM) is directly treated with T4 DNA ligase in a diluted T4 ligase buffer (e.g. 0.1× buffer), the monomeric DNA ring is prepared in a high selectivity. When necessary, the product is further treated with Exonuclease I to remove small quantity of linear byproducts, and as the result the targeted DNA ring is obtainable in a high purity which is acceptable for many applications. Among many methods proposed to date for DNA ring preparation, this combination of these two methodologies is, to the best knowledge of the authors, the most practical and advantageous in terms of simple reaction procedures and easy scale-up for preparative synthesis.

## Supplementary Material

Supplementary Data
