# Supplementary material for: Terminal hairpin in oligonucleotide dominantly prioritizes intramolecular cyclization by T4 ligase over intermolecular polymerization: an exclusive methodology for producing ssDNA rings
Source: Nucleic Acids Res. 2018 Aug 29;46(22):e132. doi: 10.1093/nar/gky769 (PMC6294566; doi:10.1093/nar/gky769)
Supplement: Supplementary Data [file gky769_supplemental_files.pdf]

Terminal hairpin in oligonucleotide dominantly prioritizes intramolecular cyclization by T4 ligase over intermolecular polymerization – An exclusive methodology for producing ssDNA rings

Yixiao Cui[1,†], Xutiange Han[1,†], Ran An[1,2,*], Yaping Zhang[1], Kai Cheng[1], Xingguo Liang[1,2,*] and Makoto Komiyama[1]

1 College of Food Science and Engineering, Ocean University of China, Qingdao 266003, China
2 Laboratory for Marine Drugs and Bioproducts, Qingdao National Laboratory for Marine Science and Technology, Qingdao 266003, China

*To whom correspondence should be addressed. Tel: +86 532 82031086; Fax: +86 532 82031086; Email: liangxg@ouc.edu.cn
Correspondence may also be addressed to Ran An. Tel: +86 532 82031318; Fax: +86 532 82031086; Email: ar@ouc.edu.cn

†These authors contributed equally to this work as first authors.

Table S1. The l-DNAs and splints used in Figures 2-7.

Name	Sequences（5′→3′）*	Length（nt）
$L64_{3-4,24-4}$	ACCGCTACACTTGCCAGCGCCCTAGCGCCCGCTCCTTTCG CTTTCTTCTCCCCATTTTCGCCGG	64
$L64_{16-4,37-4}$	CCATTTTCGCCGGACCGCTACACTTGCCAGCGCCCTAGCG CCCGCTCCTTTCGCTTTCTTCTCC	64
$L64_{3-4}$	ACCGCTACACTTGCCAGCGCCCTTTTCCCTTTCCTTTTCGCT TTCTTCTCCCCATTTTCGCCGG	64
$L74_{3-4,24-4,65-2}$	ACCGCTACACTTGCCAGCGCCCTAGCGCCCGCTCCTTTCG CTTTCTTCCCTTCCTTTCTCGCCACGTTCGCCGG	74
$L64_{3-4,24-4,55-2}$	ACCGCTACACTTGCCAGCGCCCTAGCGCCCGCTCCTTTCG CTTTCTTCTCGCCACGTTCGCCGG	64
$L54_{3-4,24-4,45-2}$	ACCGCTACACTTGCCAGCGCCCTAGCGCCCGCTCCTTTCG CTTTCGTTCGCCGG	54
$L44_{14-6,35-2}$	ACCGCTACACTTGCCAGCGCCCTAGCGCCGCTTTCGTTCG CCGG	44
$L34_{3-4,25-2}$	ACCGCTACACTTGCCAGCGCGCCACGTTCGCCGG	34
$L64_{2-4,23-4,51-2}$	CCGCTACACTTGCCAGCGCCCTAGCGCCCGCTCCTTTCGC TTTCTTCTCCCCATTTTCGCCGGA	64
$L64_{4-4,25-4}$	GACCGCTACACTTGCCAGCGCCCTAGCGCCCGCTCCTTTC GCTTTCTTCTCCCCATTTTCGCCG	64
$L64_{5-4,26-4}$	GGACCGCTACACTTGCCAGCGCCCTAGCGCCCGCTCCTTT CGCTTTCTTCTCCCCATTTTCGCC	64
$L64_{1-4,24-4}$	CGCTACACTTGCCAGCGCCCACTAGCGCCCGCTCCTTTCG CTTTCTTCTCCCCATTTTCGCCGG	64
$L64_{1-6,24-4}$	GGCGCTACACTTGCCAGCGCCCTAGCGCCCGCTCCTTTCG CTTTCTTCTCCCCATTTTCGCCGG	64
$L64_{1-7,24-4}$	GGCGCGCCACTTGCGCGCGCCCTAGCGCCCGCTCCTTTCG CTTTCTTCTCCCCATTTTCGCCGG	64
$L60_{1-7,20-4}$	GGCGCGCGAAGCGCGCCCTAGCGCCCGCTCCTTTCGCTTT CTTCTCCCCATTTTCGCCGG	60
$L64_{6-4,27-4}$	CGGACCGCTACACTTGCCAGCGCCCTAGCGCCCGCTCCTT TCGCTTTCTTCTCCCCATTTTCGC	64
$Splint_{3-4}$	AGCGGTCCGGCG	12
$Splint_{16-4}$	AAATGGGGAGAA	12
$Splint_{2-4}$	TAGCGGTCCGGC	12
$Splint_{4-4}$	GCGGTCCGGCGA	12
$Splint_{5-4}$	CGGTCCGGCGAA	12
$Splint_{1-4}$	GTAGCGCCGGCG	12
$Splint_{1-6}$	AGCGCCCCGGCG	12
$Splint_{1-7}$	CGCGCCCCGGCG	12
$Splint_{6-4}$	GGTCCGGCGAAA	12

*The underlined parts ("_") of l-DNA are complementary with splint.

Table S2. The percentage of l-DNAs with stable haipin(s) calculated by Mfold

The length of l-DNA*	50 nt		100 nt	
Hairpin quantity	One or more	two or more	one or more	two or more
Percentage with stable hairpin (T_m >50°C)	39%	6%	69%	14%

*Human genome (ADAM) is used as the source of sequences, and the number of used l-DNA sequences for checking secondary structures is 100. Only the hairpin with a T_m higher than 50°C was counted. Percentage with stable hairpin means the percentage of l-DNAs with stable hairpin(s) in all the l-DNA we tested.

The conditions: [Mg^{2+}] = 10 mM and 25°C.

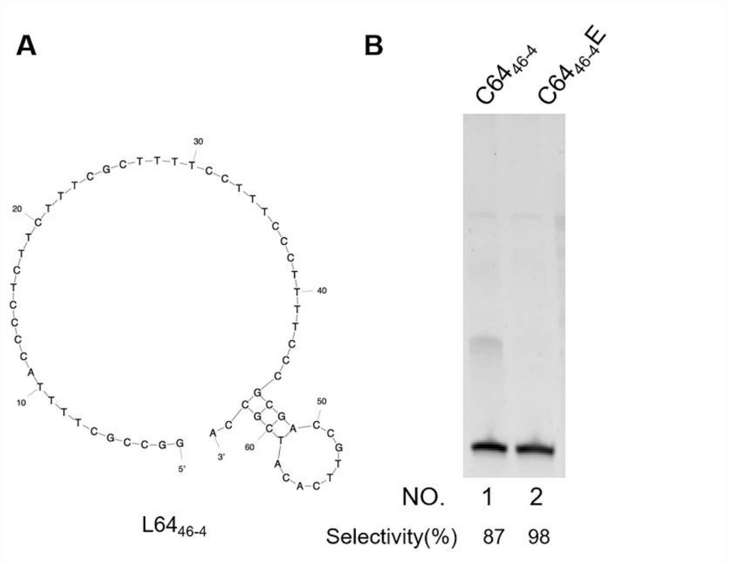

Figure S1. Effects of hairpins on the cyclization of l-DNA. In (A), the solution structures of L64$_{46-4}$ determined by Mfold calculation are shown. In (B), these l-DNAs were cyclized by T4 DNA ligase. Lane 1, L64$_{46-4}$ treated with T4 DNA ligase in the presence of 12-nt splint; lane 2, the products in lane 1 treated with Exonuclease I to remove non-cyclic products. The conditions: [L64$_{46-4}$]= 5 μM, [Splint$_{46-4}$]= 10 μM, and 10 U T4 DNA ligase in 1× T4 DNA ligase buffer at 25°C for 12 h.

Sequences of oligonucleotides used here are as follows:

L64$_{46-4}$: GGCCGCTTTTACCCCTCTTCTTTCGCTTTTCCTTTCCCTTTTCCCGCGACCGTTCACATCGCCA

Splint$_{46-4}$: GCGGCCTGGCGA

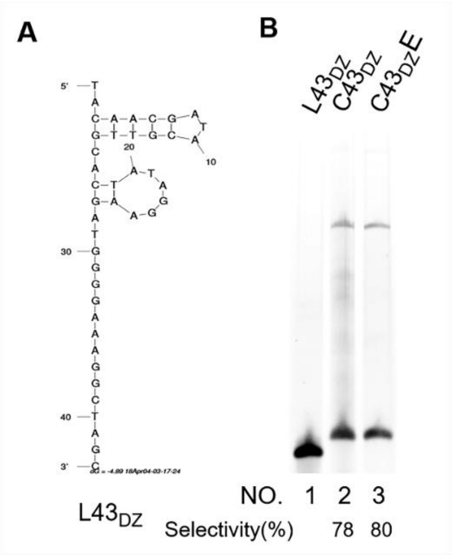

Figure S2. Cyclization of a 10-23 DNAzyme (L43$_{DZ}$). (A) The solution structures of L43$_{DZ}$ determined by Mfold calculation under the conditions of [Mg^{2+}] = 10 mM and 25°C. (B) Treatments of L43$_{DZ}$ with T4 DNA ligase. Lane 1, L43$_{DZ}$ without the T4 ligase treatment; lane 2, L43$_{DZ}$ treated with T4 DNA ligase in the presence of 12-nt splint; lane 3, the products in lane 2 treated with Exonuclease I. The conditions for the cyclization: [L43$_{DZ}$]$_0$= 5 μM, [Splint $_{DZ}$]$_0$= 10 μM, and 10 U T4 DNA ligase in 1× T4 DNA ligase buffer at 25°C for 12 h.

Sequences of oligonucleotides used here are as follows:

L43$_{DZ}$: TACAACGATACGTTGCACTATAGGAAGATGGGGAAAGGCTAGC

Splint$_{DZ}$: TCGTTGTAGCTAGCCT

The DNAzyme function of L43$_{DZ}$ was 10-23 (reported in Wang,B., Cao,L., Chiuman,W., Li,Y. and Xi,Z. (2010) Probing the function of nucleotides in the catalytic cores of the 8−17 and 10−23 DNAzymes by abasic nucleotide and C3 spacer substitutions. *Biochemistry*, **49**, 7553-7562.).

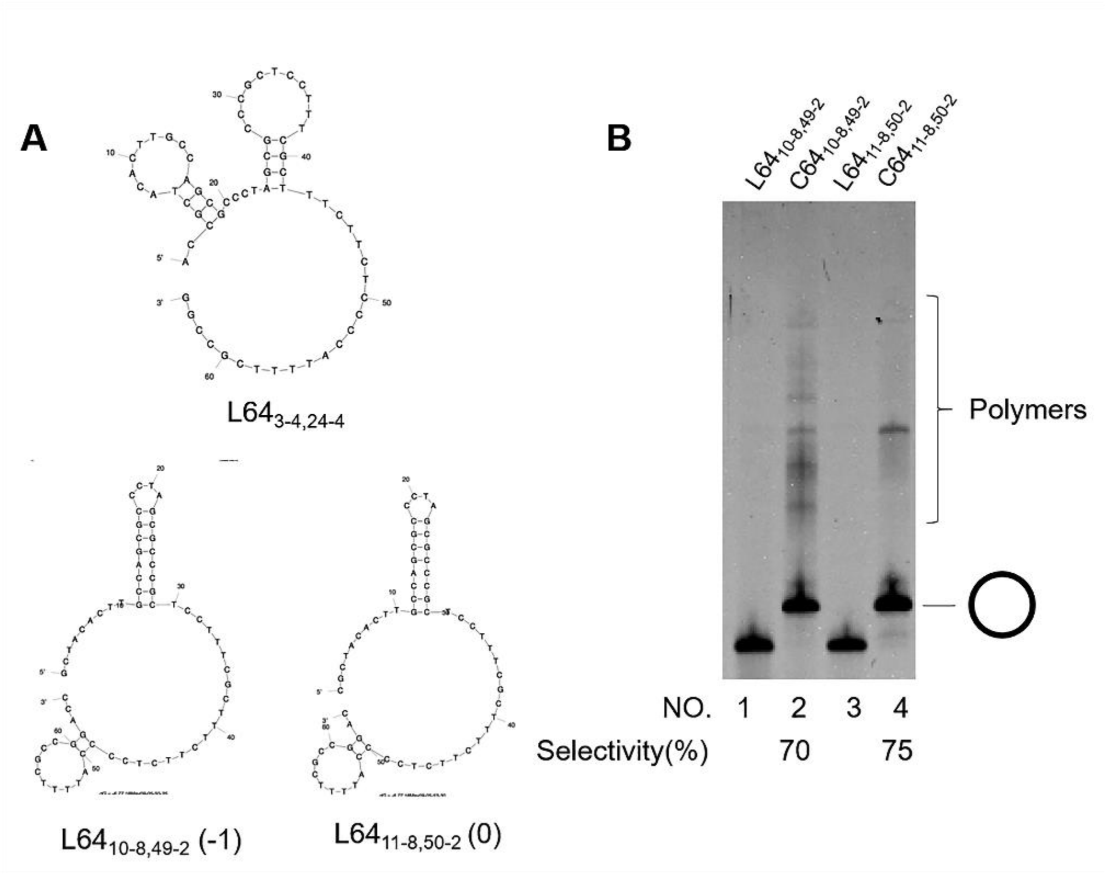

Figure S3. Cyclization of $L64_{10\text{-}8,49\text{-}2}$ and $L64_{11\text{-}8,50\text{-}2}$. (A) The solution structures of $L64_{10\text{-}8,49\text{-}2}$ and $L64_{11\text{-}8,50\text{-}2}$ determined by Mfold calculation. For the purpose of comparison, the structure of $L64_{3\text{-}4,24\text{-}4}$ is also shown. (B) Lane 1, $L64_{10\text{-}8,49\text{-}2}$ without the T4 ligase treatment; lane 2, $L64_{10\text{-}8,49\text{-}2}$ treated with T4 DNA ligase in the presence of 12-nt splint; lane 3, $L64_{11\text{-}8,50\text{-}2}$ without the T4 ligase treatment; lane 4, $L64_{11\text{-}8,50\text{-}2}$ treated with T4 DNA ligase in the presence of 12-nt splint. The conditions for the cyclization: $[\text{l-DNA}]_0$= 5 μM, $[\text{splint}]_0$= 10 μM, and 10 U T4 DNA ligase in 1× T4 DNA ligase buffer at 25°C for 12 h.

Sequences of oligonucleotides used here are shown as follows:

$L64_{10\text{-}8,49\text{-}2}$:

GCTACACTTGCCAGCGCCCTAGCGCCCGCTCCTTTCGCTTTCTTCTCCCCATTTTCGCCGGACC

$L64_{11\text{-}8,50\text{-}2}$:

CGCTACACTTGCCAGCGCCCTAGCGCCCGCTCCTTTCGCTTTCTTCTCCCCATTTTCGCCGGAC

$Splint_{10\text{-}8}$: TGTAGCGGTCCG

$Splint_{11\text{-}8}$: GTAGCGGTCCGG

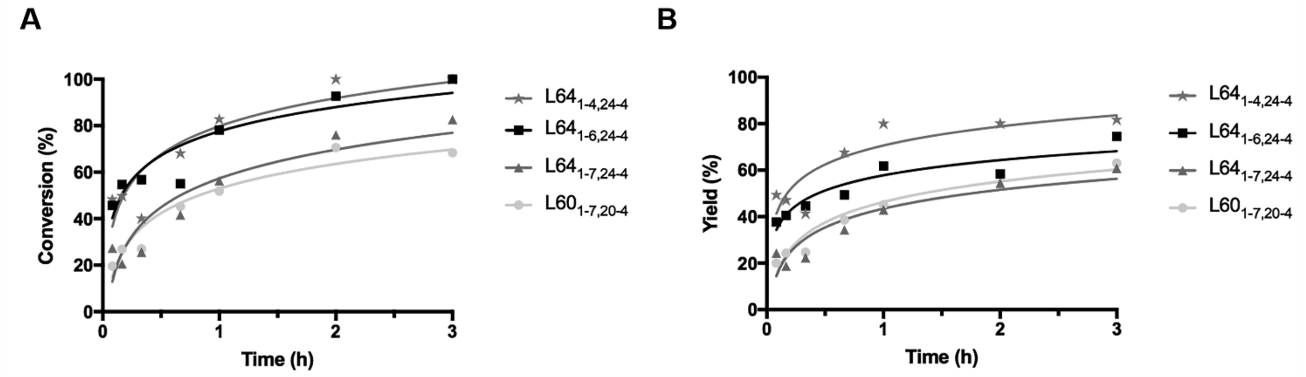

Figure S4. Comparison of the reaction conversion and yield of monomeric cyclic ring by T4 ligase. (A) The total amounts of DNA, consumed in the presence of T4 (by both intramolecular and intermolecular ligation), are plotted as a function of reaction time. In (B), the yield of DNA ring is shown as a function of reaction time. Reaction conditions: $[\text{l-DNA}]_0$ = 5 μM, $[\text{splint}]_0$ = 10 μM, and 10 U T4 DNA ligase in 1× T4 DNA ligase buffer, 25°C.

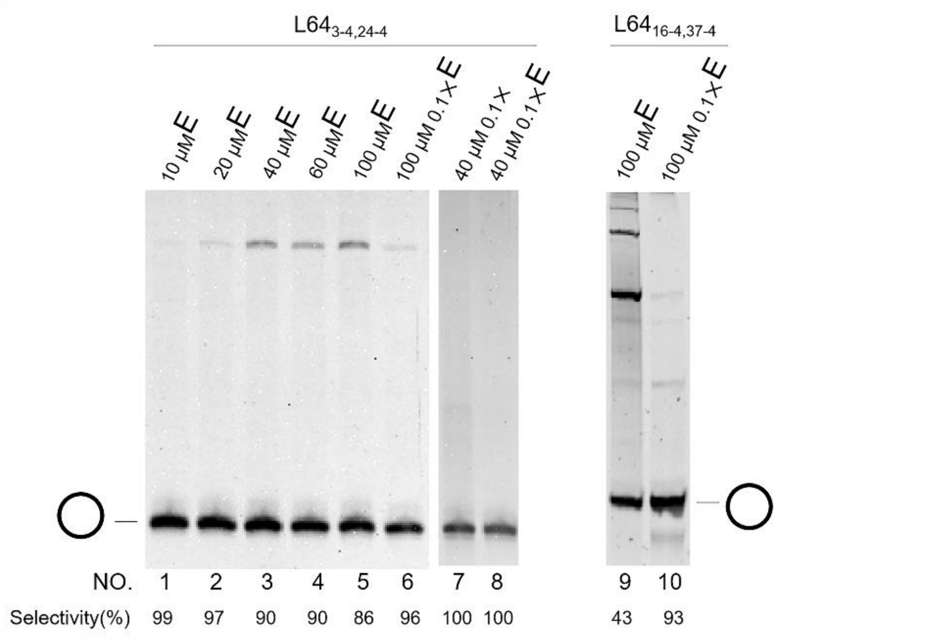

Figure S5. Combination of terminal hairpin strategy with diluted buffer strategy for highly selective cyclization in preparative scale. Lane 1, T4 reaction at [L64$_{3-4,24-4}$] = 10 μM with Exonuclease I in 1× T4 DNA ligase buffer; lane 2, 20 μM; lane 3, 40 μM; lane 4, 60 μM; lane 5, 100 μM; lane 6, [L64$_{3-4,24-4}$] = 10 μM with Exonuclease I , 0.1× T4 DNA ligase buffer was used in place of 1× T4 buffer; In lane 7, [L64$_{3-4,24-4}$] = 40 μM in 0.1× T4 DNA ligase buffer; lane 8, the products in lane 7 with Exonuclease I; lane 9, L64$_{16-4,37-4}$ with no terminal hairpin was used, [L64$_{16-4,37-4}$] = 100 μM with 1× T4 DNA ligase buffer; lane 10, [L64$_{16-4,37-4}$] = 100 μM with 0.1× T4 DNA ligase buffer. [l-DNA]$_0$/[splint]$_0$ =1/2, and 10 U T4 DNA ligase 25°C.

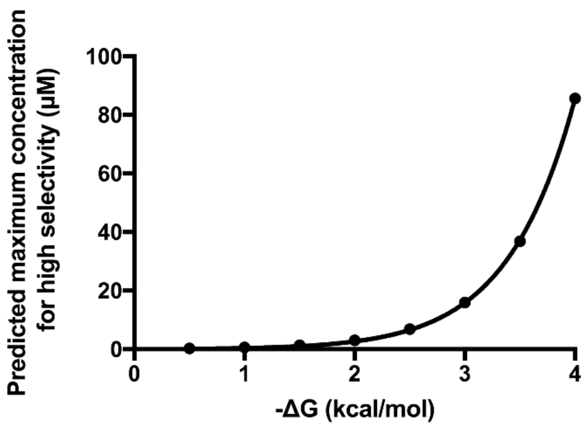

Figure S6. Predicted maximum concentration for high selectivity (>85%) by ΔG (the concentration of the active species is set as 0.1 μM). The Equilibrium constant (*K*) and concentration of hairpin structure and its linear structure was calculated by equations (1) and (2):

$\Delta G = -RT\ln K$ (1)

$K = e^{-\frac{\Delta G}{RT}} = \frac{C_{max} - C_{linear}}{C_{linear}}$ (2)
